# Association of Clinical, Biomechanical, and Psychosocial Factors with Smile Dynamics in Unilateral Cleft Lip: A Multicenter Observational Study

**DOI:** 10.1177/10556656241291649

**Published:** 2024-12-05

**Authors:** Lucinda Wong, Fiona Firth, Peter Fowler, Hannah Jack, Hamza Bennani, Thomas Noble Campbell, Mauro Farella

**Affiliations:** 1 Discipline of Orthodontics, Faculty of Dentistry, 2495 University of Otago, Dunedin, New Zealand; 2 Department of Surgical Sciences, University of Cagliari, Cagliari, Italy

**Keywords:** artificial intelligence, assessment, cleft lip, lip function, orthodontics, quality of life, young adults, scarring

## Abstract

**Objective:**

To investigate the association between clinical, biomechanical, and psychosocial factors and smiling behavior in individuals with treated unilateral cleft lip with or without cleft palate (UCL ± P) compared to non-cleft controls.

**Design:**

Multicenter observational study in New Zealand.

**Participants:**

Individuals aged ≥15 (*N* = 42) comprised 2 study groups: a UCL ± P group (*N* = 21) and a non-cleft control group (*N* = 21).

**Methods:**

Participants viewed an amusing video while their facial expressions were recorded. Smile features were automatically detected via software. A clinical outcome, nasolabial esthetics, was scored using the Asher-McDade system. Perioral biomechanical properties were measured via myotonometry. Smile Esthetics-related Quality of Life (SERQoL), Orofacial Esthetics Scale (OES), and personality (IPIP-NEO-60) questionnaires were completed.

**Results:**

Smile features and personality traits did not differ between the groups. Participants with UCL ± P exhibited higher stiffness (+44.2%; Cohen's *d *= 1.6) and tone (+22.6%; Cohen's *d* = 1.9) at the cleft scar site, and higher decrement (or lower elasticity, +8.5%; Cohen's *d *= 0.8) adjacent to the scar. Nasolabial esthetics and elasticity of the scar correlated with the duration of smiles and relative smile time (−0.50 < R < −0.44; *p* < .05). Participants in the UCL ± P group had lower scores for the OES and higher impacts on SERQoL for social contacts and dental self-confidence.

**Conclusions:**

Adolescents and adults with UCL ± P exhibit similar smile behavior as their cleft-free peers—at least in non-social settings. Nasolabial esthetics and perioral biomechanical properties are associated with propensity to smile. UCL ± P is negatively associated with smile-related quality of life and an individual's perception of their facial appearance.

## Introduction

Individuals with cleft lip and/or palate (CL/P) undergo a comprehensive treatment journey. The course of care varies greatly depending on the diagnosis, which can range from an isolated notch in the lip or uvula, through to a full separation of the lip and palate extending though alveolar bone and into the nasal cavity. Treatment requires a multidisciplinary approach involving both surgical and non-surgical interventions. While the primary goal is to restore orofacial function and appearance, in many cases it is unavoidable that certain remnants of the cleft, such as scar tissue and altered smile esthetics, may be visible even after treatment is complete, particularly when the lip is affected.

People with CL/P may face ongoing functional, esthetic, and psychosocial challenges.^1–3^ While overall psychological outcomes appear to be similar between children with CL/P and their non-cleft peers, symptoms such as anxiety and depression are more prevalent in the former group.^4^ These issues may arise from experiences of bullying about cleft-related features, dissatisfaction with facial appearance, speech difficulties, reduced self-confidence, and emotional distress.^5–8^ Such challenges may lead to lasting behavioral adjustments and impact social interactions.^5,9^ Additionally, the impact of CL/P extends beyond treatment completion, negatively affecting health-related quality of life in adults with CL/P, particularly in psychosocial dimensions.^10,11^

Smiling, a crucial form of non-verbal communication,^12^ is self-reported to be less frequent and expressive among individuals with CL/P.^5^ Objective research on cleft smiles has predominantly assessed outcomes such as facial landmark displacement and smile asymmetry.^13–17^ A recent study has also delved into the physical impact of soft tissues and discovered that stiffer cleft scars can impair the generation of facial expressions.^17^ However, there remains a lack of research that objectively measures how dynamic features of spontaneous smiles may vary in individuals with CL/P from a psychosocial perspective, particularly regarding their inclination to smile.

In this study we are using an automated smile detection software previously created by our research team. This software was developed using the Facial Action Coding System (FACS), which attributes different facial expressions to muscular movements.^18,19^ Smiling that depicts situations of pure enjoyment or laughter, is often referred to as a genuine smile or ‘Duchenne smile’, and elicits combined activation of the *zygomaticus major* and *orbicularis oculi* muscles.^20–22^ This software is able to accurately identify smile frequency, duration, intensity, tooth display, and to some extent, also smile genuineness.^18^

This study aims to examine the association between unilateral cleft lip with or without cleft palate (UCL ± P) and smile behavior in older adolescents and adults who have completed their treatment. Additionally, we aim to explore the relationship between clinical, biomechanical, and psychosocial factors with one's inclination to smile. We hypothesise that individuals with UCL ± P will smile less frequently and for shorter duration compared to their cleft-free counterparts. Furthermore, we anticipate that unsatisfactory nasolabial esthetics, stiff and non-elastic lip musculature, and negative self-reported orofacial esthetics and smile esthetics related quality of life, will be associated with reduced smile frequency, duration, intensity and tooth display.

## Materials and Methods

This report follows the Strengthening the Reporting of Observational Studies in Epidemiology (STROBE) guidelines.^23^

### Study Design and Setting

A multi-center, matched case-control observational study was conducted across 4 locations in New Zealand: Dunedin, Wellington, Christchurch, and Auckland. The study was carried out at the Faculty of Dentistry Craniofacial Research Laboratory and Auckland Dental Facility, the Hutt Valley District Health Board Dental Department, and a private dental clinic in Christchurch. Recruitment and data collection were carried out simultaneously during a 1-year period spanning from March 2022 to March 2023.

### Ethical Approval and Funding

Ethical approval was granted from the University of Otago Human Ethics Committee (reference: H22/008). Prior to the investigation all study participants were provided with information about the study and signed written informed consent.

*Sample size estimation* The sample size calculation was based on previous data on the frequency and variability of smiles, with the activation of the lip puller muscle as the main outcome variable.^20^ Twenty-one participants in each group will give the study a power of 80% to detect a meaningful difference of 50% in the frequency of smiling between the groups (alpha = .05).

### Participant Inclusion/Exclusion Criteria and Recruitment

Forty-two participants were recruited, matched, and allocated to 1 of 2 study groups: the cleft group (*N* = 21) and the non-cleft control group (*N *= 21). Participants included in the cleft group were people with UCL ± P, aged 15 years or above. Participants must have completed all cleft lip-related surgeries and active orthodontic treatment at the time of recruitment. Those in the retention phase of orthodontic treatment, or awaiting other cleft surgeries unrelated to the lip (eg, rhinoplasty) were also eligible. Exclusion criteria were: diagnosed syndromes; Bell's palsy; current self-reported depression; and psychiatric disorders. Additionally, participants were requested to be clean-shaven to facilitate the scoring of a nasolabial clinical outcome.

The study did not include individuals with cleft palate alone because we anticipated greater variations in smiling characteristics would be observed when the lip is affected. We also excluded those with bilateral clefts due to the low incidence of this condition and the potential complexity it would introduce during data analysis.

Recruitment for participants in the cleft group was achieved by contacting previous patients of the University of Otago Post-graduate Orthodontic Clinic and Hutt Valley Dental Department, social media posts and email advertisements circulated by Cleft New Zealand (a cleft support organisation), and word of mouth.

Participants in the non-cleft control group were individually matched for age (± 2 years), sex, and ethnicity. Whenever possible, they were recruited based on suggestion of a friend or family member of each person in the cleft group. Prior to data collection, all participants underwent eligibility screening (Appendix 1).

### Assessments

#### Video Recording

Each participant was seated in a room and left unsupervised to watch an amusing video, used to elicit smiling reactions. Facial expressions were captured using a high-definition web camera (Logitech Brio 4 K) mounted above the video display screen (27-inch Dell monitor). The camera was set to a resolution of 4096 × 2160 pixels, capturing 30 frames per second.

Participants were seated 60 cm from the display screen, and the height of the monitor was adjusted to eye-level. A chromakey green screen or a black screen was used as the background, depending on the natural lighting conditions of the room. The room lights were on, and an additional light source (Godox SL-60 LED) was placed to the side of the screen to create light reflection from the walls onto the participants’ faces. The camera exposure settings were adjusted to each participant, and videos were recorded using the Logitech Capture software.

The smile-triggering video was 6 min long and consisted of a compilation of 8 amusing videos. Among these, 3 videos had been validated and used in previous research: Mr Bean, a cute giggling baby, and Juan Joya Borja's viral laughing video.^18,24^ The remaining 5 videos were a series of social platform reels, which were selected out of 10 clips chosen by LW. These clips were presented individually to a convenience sample of 10 participants located in Dunedin and Wellington, who represented diverse demographics in terms of age (32 ± 16 years), sex (6 females; 4 males), and ethnicity (8 Europeans, 1 Asian, and 1 Māori). The final selection of the 5 video clips was based on the feedback gathered. The inclusion of these additional video clips, alongside the previously validated ones, aimed to accommodate a more diverse range of humor.

A second video segment, lasting for a duration of 7.5 min, was shown after the smile-triggering video. This segment involved a series of tasks in which participants were instructed to perform actions, such as displaying posed emotions, counting, and yawning. These tasks were used to calibrate the smile detection software algorithm and identifying any expressions that may introduce confounding factors. The full video stimuli and examples of participant reactions are available on request.

#### Smile Analysis

Smile characteristics were the primary outcome of this study and were generated using a previously validated method of smile analysis.^18^ This involved analysis of participants’ video recordings frame by frame using the open-source FACS automated software (OpenFace 2.2.0). The software tracks facial landmarks associated with the Facial Action Coding System (FACS)^19^ and measures the activation of specific action units (AU) relevant to smiling, namely AU6 (Duchenne marker, cheek raiser—*orbicularis oculi, pars orbitalis*), AU12 (lip corner puller—*zygomaticus major*), and AU25 (lips apart-corner puller—*depressor labii*).

The action unit data was then processed using a custom-made script to detect smile episodes. A smile episode was characterised by the activation and deactivation of muscle action units AU6 and AU12, surpassing predefined thresholds of 0.5 and 1.5, respectively. The termination of a smile episode was determined when either of the action units remained activated below the threshold for more than 1 s. The software calculated the start time, duration, and mean activation levels of AU6 and AU12 for each smile episode. To provide a clinically relevant measure for AU25, it was reported as the percentage of time during a specific smile episode that teeth were visible.

These values were used to calculate the frequency of smiles (number of episodes per minute), the mean duration of smiles, the mean activation (ranging 0-5) of AU6 and AU12, and the mean percentage activation of AU25 across all smiles. Additionally, the relative smile time percentage was calculated by dividing the total duration of all smile episodes by the length of the amusing video, providing a measure of the proportion of time spent smiling.

The final 6 smile outcomes analyzed in this study were: frequency of smiles, mean duration of smiles, relative smile time (%), smile genuineness (mean activation of AU6), smile intensity (mean activation of AU12), and the amount of tooth show, which was indirectly measured by the distance between the lips (mean percentage activation of AU25).

### Cleft Clinical Outcome

A cleft clinical outcome was assessed using a well-established and validated nasolabial scoring system,^25^ the Asher-McDade (AM) score. Facial photographs of participants in the cleft group were taken in the in the frontal and lateral (left and right) views.

AM scoring was conducted over 2 sessions by a panel of 3 examiners—2 orthodontists and 1 pediatric dentist. To maintain blinding, none of the examiners were involved in the data collection process. Prior to scoring, a calibration session was conducted to establish a consensus among the examiners regarding the nasolabial esthetic scores. This session involved evaluating 10 sets of photos featuring individuals with unilateral cleft lip who were not part of the study. The examiners were provided with a set of reference photos to assist in the scoring process.^26^

Four nasolabial features were assessed: nasal form, nasal symmetry, vermillion border, and nasal profile. A 5-point Likert scale was used to rate the appearance of each feature, with 1 indicating “very good” and 5 indicating “very poor.”

A frontal and lateral image of each cleft group participant was displayed on a PowerPoint presentation. Images were cropped to show only the nasolabial area, therefore minimizing bias from surrounding features. The photos of participants with right-side clefts were horizontally flipped, so that all images appeared to have left-side clefts. The photo sets were displayed one at a time in a random order, while the examiners independently scored them. No discussion among examiners was allowed, and each slide remained on display until all examiners had completed scoring. A black slide was shown between each photo set to reduce influence from the previous image.

The scoring session was repeated 1 week later with the same photo sets presented in a different random order to enable the calculation of intra-examiner reliability. The mean scores across the 2 sessions and between the 3 examiners were calculated to give a final score for each of the 4 nasolabial components. The mean score across all components was then calculated to obtain an overall nasolabial score ranging from 1 (best possible score) to 5 (worst possible score) for each participant.

### Biomechanical Perioral Properties

Biomechanical characteristics of the perioral muscles and cleft lip scar, or the equivalent site for non-cleft participants, were measured. Participants were seated on a dental chair and adjusted to a supine position. They were instructed to have their teeth lightly in occlusion and lips relaxed. The perioral musculature was assessed using a recently validated method of myotonometry.^27^ A handheld digital palpation device (Myoton MPRO, Myoton AS, Tallin, Estonia) was used at 5 designated sites surrounding the lips. In addition to the established method, an additional site, namely “Cleft Scar,” was included. The location of each landmark was determined with a ruler and marked using a pen. To mitigate systematic error, the 5 landmark locations were assessed using random sequences in balanced blocks.

The Myoton device comprises an accelerometer linked to a 10 mm probe. A force of 0.58 N is applied perpendicular to the skin and the Myoton produces a brief mechanical pulse. Five consecutive pulses lasting 15 ms and spaced 0.8 s apart, were recorded. If the coefficient of variation between the pulses was greater than 3%, the measurement was retaken. Measurements were repeated 3 times at each of the 5 landmark locations (Appendix 2).

The Myoton device generated 3 outcome measures: tone, stiffness, and decrement, which represent biomechanical properties of the tissue. Tone was quantified using oscillation frequency (Hz), stiffness is a measure of tissue deformation under a given force (Nm), and decrement is determined as the rate of decline in oscillation amplitude following tissue displacement (arbitrary units). Decrement is inversely related to tissue elasticity, meaning that a higher decrement value indicates lower tissue elasticity. A tissue with low elasticity has a reduced capacity to stretch under applied force and return to its original shape once the force is removed.

The values of the 3 readings taken were averaged to obtain the mean tone, stiffness, and decrement at each of the 5 perioral sites.

### Questionnaire Data

Smile Esthetics-related Quality of Life, Orofacial Esthetics Scale, & Personality Questionnaires were collected using either an iPad tablet or the participants’ personal smart phone.

The SERQoL questionnaire comprises 12 questions that assess 3 dimensions of psychosocial impacts associated with altered smile esthetics.^28^ Participants rated their responses using a Likert 5-point scale, where 1 corresponds to “strongly disagree” and 5 corresponds to “strongly agree.” Three outcome scores were generated from the SERQoL survey: dental self-consciousness (DSCf), social contacts (SC), and dental self-confidence (DSCs). A high score for DSCf suggests that an individual exhibits low self-consciousness regarding their smile (favorable), while high scores for SC and DSCs indicate that a person perceives their smile as having a negative impact on their social life and possesses lower self-confidence concerning their smile, respectively (unfavorable).

The OES questionnaire consists of 8 questions and is employed to evaluate individuals’ self-perceived dental and facial attractiveness.^29,30^ It employs an 11-point numerical scale, where 0 indicates “dissatisfied” and 10 represents “very satisfied.” Questions 1 to 7 of the survey aim to evaluate satisfaction with specific aspects: the appearance of the face, profile, mouth, tooth alignment, tooth shape, tooth color, and gums. The 8th question measures participants’ overall perception of their orofacial appearance and can serve as a validation for the combined responses of questions 1 to 7 or as a standalone assessment.^31^ Each aspect of orofacial appearance (questions 1-7) were evaluated individually. In addition, the scores for questions 1 to 7 were combined to generate an OES total score, while OES question 8 was assessed separately. Therefore, a total of 9 scores were produced from the OES.

The IPIP-NEO-60 questionnaire consists of 60 items that evenly cover the 5 major personality traits: openness, conscientiousness, extraversion, agreeableness, and neuroticism.^32^ Participants responded to scenario-based questions using a 5-point Likert scale, where 1 represents “strongly disagree” and 5 represents “strongly agree.” The IPIP-NEO-60 questionnaire yielded 5 scores, representing personality traits, with each trait score ranging from 12 to 60. Since personality can be a confounding factor in relation to smiling behavior, this questionnaire was primarily utilised to allow for appropriate adjustments to be made if the personality traits varied between the 2 study groups.

### Study Procedure

Expressions of interest were gathered during the recruitment process and eligibility screening was completed via a phone survey or email. Those who were eligible and willing to partake were booked a 1-hour session at one of the 4 study locations. Participants were emailed the study information and consent forms to complete online prior to their study session date. Study sessions in Wellington, Auckland, and Christchurch took place during specific limited dates because a member of the study team needed to travel to these locations; whereas data collection in Dunedin was able to take place on any date that was suitable for the participant.

Full sets of data for each participant were collected once by 1 of 2 members of the research team. With the aim of minimizing inter-rater bias, the data for each matched cleft and control pair were collected by the same person in all cases except for 3 instances.

Participants underwent data collection individually during a single session. On arrival, participants were given a brief overview of the study proceedings. Their details, eligibility, and consent forms were double checked, and time was allowed for any questions. The location of treatment for those in the cleft group was also recorded. Data were then collected in the following sequence: video recording, SERQoL and OES questionnaires, facial and intraoral photographs (for cleft-group participants only), biomechanical measurements of the lips, and lastly the IPIP-NEO-60 questionnaire. The purpose of the intraoral photographs was to validate the cleft diagnosis, given that some participants had uncertainty about their diagnosis.

Participants were deliberately not informed about the study's focus on smiling prior to watching the video, as this approach was deemed essential to capture authentic and spontaneous smiling reactions. The 3 questionnaires were divided into 2 at the beginning of the session and 1 at the end to avoid survey fatigue.

Following data collection, participants were informed about the specific focus of the study, which was to explore the influence of cleft lip conditions on smiling behavior. Ample time was provided for participants to ask questions and engage in discussions related to the study. Additionally, participants were requested to rate the amusing video on a scale ranging from 0 (not funny at all) to 10 (hilarious). As compensation for their time and travel expenses, each participant received an $80 supermarket voucher.

### Statistical Analysis

All data was deidentified and encrypted to ensure participants’ confidentiality, and then analysed using the Statistical Package for the Social Sciences (SPSS 28.0, IBM, Chicago, Illinois, USA). The Kolmogorov-Smirnov test was used to check if the data followed a normal distribution. Depending on the outcome of the normality test, comparisons were performed using either the paired Student's t-test or the non-parametric Wilcoxon Pratt test, while correlation analysis was computed using either the Pearson or Spearman correlation coefficient. Smile data was analysed using mixed model analysis. Effect sizes were assessed using Cohen's d. Intra-rater and inter-rater reliability were assessed using intraclass correlation coefficients. Statistical significance was accepted for p-values less than 0.05.

## Results

### Demographic Characteristics

The study participants had an age range of 16 to 63 years, with a median age of 24 years, and approximately half were females (Table 1). Most participants were of European ethnicity, and 2 participants of Chinese ethnicity. In the cleft group, most participants were located in Dunedin (7) and Wellington (7), followed by Christchurch (4), and Auckland (3). In the non-cleft control group, the number of participants in Dunedin was higher (12), equal in Christchurch (4), and fewer in Wellington (3) and Auckland (2). Most of the clefts involved both the lip and palate (81%), and 76% were located on the left-side.

**Table 1.** Demographic Information of Study Participants.

	Cleft group (*N* = 21)	Control group (*N* = 21)
Age, years	28 ± 12	28 ± 13
*Gender*		
Female	10 (48)	10 (48)
Male	11 (52)	11 (52)
*Ethnicity*		
Caucasian	20 (95)	20 (95)
Asian	1 (5)	1 (5)
*Location*		
Dunedin	7 (33)	12 (57)
Wellington	7 (33)	3 (14)
Christchurch	4 (19)	4 (19)
Auckland	3 (14)	2 (10)
*Cleft type*		
Cleft lip and palate	17 (81)	
Cleft lip	4 (19)	
*Cleft laterality*		
Left	16 (76)	
Right	5 (24)	

*Note*: Values are presented as *M* ± SD or *n* (column %).

### Smile Outcomes

Frequency of smiles, mean duration of smiles, relative smile time percentage, smile genuineness (AU 6), smile intensity (AU 12), and tooth show (AU 25) did not differ significantly between the cleft and the non-cleft groups (Appendix 3). The mean activation of the action units was calculated after excluding 2 participants who did not smile at any stage during the video - both of whom were from the cleft group. Data from their matched non-cleft participants were also excluded. The large error bar for mean duration of smiles in the non-cleft group was due to an outlier who smiled for most of the video. A new statistical comparison was run after removing this outlier, along with their matched cleft group participant, and the difference in mean duration of smiles was still not statistically significant (Wilcoxon Pratt test; p = 0.82).

### Cleft Clinical Outcome (AM Score)

The overall nasolabial AM scores ranged from 1.3 to 3.6, with a mean score (± SD) of 2.5 ± 0.6. The mean scores for each component were: 2.8 ± 1.1 for nasal form, 2.4 ± 0.8 for nasal symmetry, 2.3 ± 0.8 for vermillion border, and 2.4 ± 0.9 for nasal profile. The inter-rater reliability was good (ICC = 0.87) and the intra-rater reliability was excellent (ICC = 0.93). The overall mean nasolabial AM score was slightly higher for participants with a unilateral cleft of the lip and palate (UCLP) (2.6 ± 0.6), compared to participants with a unilateral cleft of the lip alone (UCL) (2.1 ± 0.5). The difference was not statistically significant (*p* = .167).

### Biomechanical Properties of the Lips

**Figure 1.**
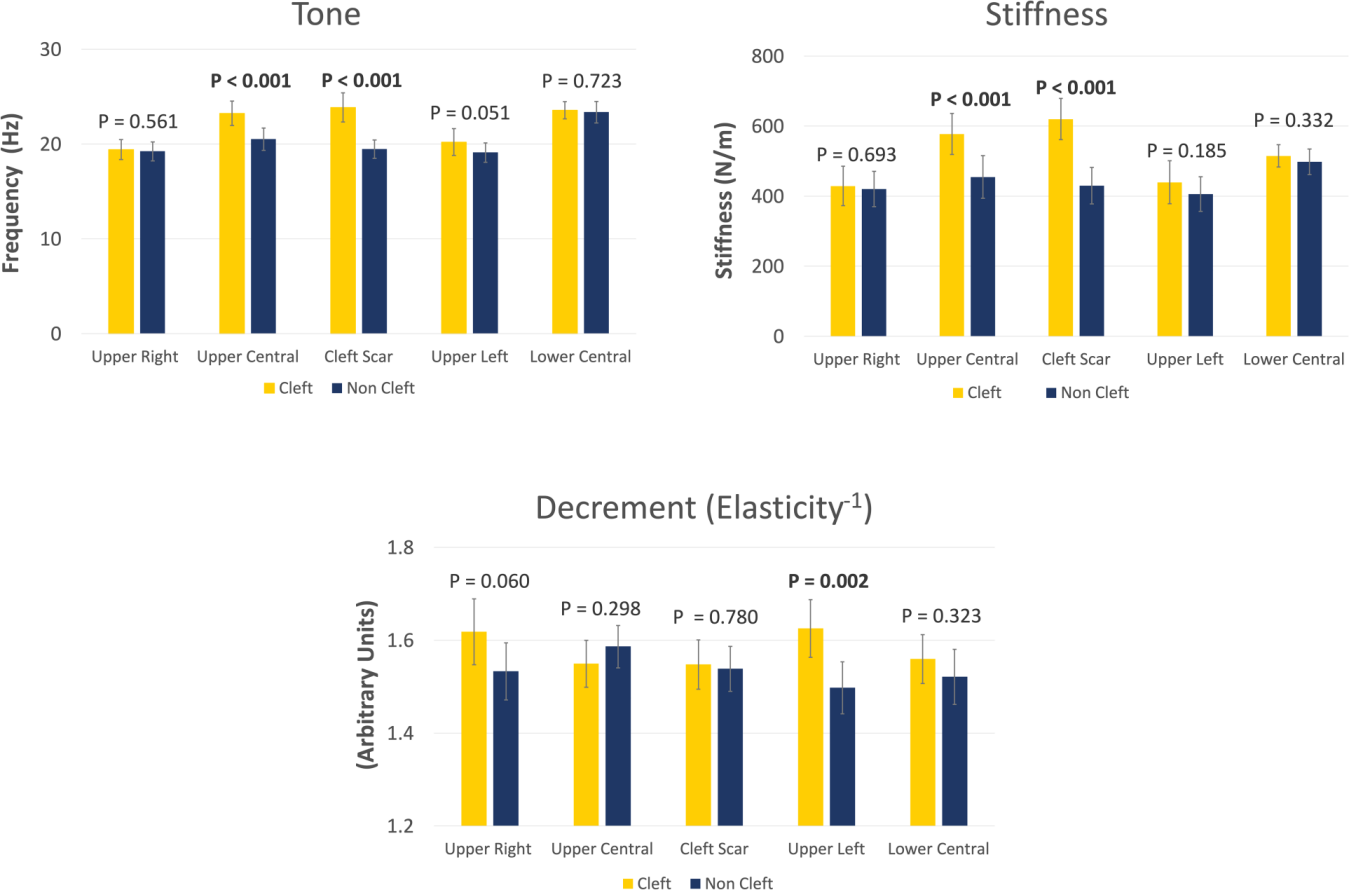
Bar graphs showing the biomechanical properties of tone, stiffness, and decrement (elasticity-1) at the five sites around the lips in the 2 study groups. Vertical bars represent mean values of each group, whereas error bars represent 95% confidence intervals. Values were compared using the paired student t-test. The group comparison *p*-values are stated above the bars, with statistically significant *p*-values indicated in bold.

The cleft group exhibited higher lip tone and stiffness at both the cleft scar point (+22.6% tone; +44.2% stiffness) and the upper central lip point (+13.3% tone; +27.0% stiffness) compared to the non-cleft controls (Figure 1). These differences were highly statistically significant (*p* < .001) and with a large effect size (Cohen's *d* > 0.8). Additionally, the cleft group showed elevated lip tone (+5.8%) at the upper left lip point, although the difference between the groups only reached marginal statistical significance (*p* = .051).

In terms of decrement values, the cleft group had higher values (+8.5%) at the upper left lip site, indicating that this area of the lip was less elastic compared to the non-cleft group. This finding was highly statistically significant (*p* = .002) and with a substantial effect size (Cohen's *d* = 0.8). Additionally, higher decrement (+5.5%) was observed in the cleft group at the upper right lip; however, the difference between the cleft and non-cleft groups only approached statistical significance (*p* = .060).

A schematic representation of the biomechanical properties of perioral musculature in cleft group participants, as compared to non-cleft controls, is provided in Appendix 4. To facilitate the interpretation of these properties, the concept of decrement was translated into elasticity, as they are inversely related; therefore, as the decrement values increased, the elasticity values decreased. No statistically significant differences between the study groups were found for any of the biomechanical properties at the lower lip.

### Smile Esthetics-Related Quality of Life and Orofacial Esthetics Scale

Participants in the cleft group had worse scores for smile esthetics-related quality of life in the categories of social contacts (SC) and dental self-confidence (DSCs) (Table 2). The cleft group also reported lower global and total scores for their perceived facial appearance (OES). Of the 7 subset questions in the orofacial esthetics scale, participants in the cleft group scored lower in 6 of them, with the exception being question 6, which was regarding the color of teeth.

**Table 2.** Smile Esthetics-Related Quality of Life (SERQoL) and Orofacial Esthetics Scale (OES) Scores.

	Cleft group (*N* = 21)	Control group (*N* = 21)	Cohen's *d*	*p*
*SERQoL scores*				
Dental self-consciousness (DSCf)	13.1 ± 4.0	14.9 ± 3.1	0.32	.153
Social contacts (SC)	7.1 ± 3.1	4.9 ± 1.6	0.76	.**002**
Dental self-confidence (DSCs)	9.5 ± 3.9	5.8 ± 2.1	0.76	.**002**
*OES scores*				
Global score (Q8)	6.2 ± 1.6	7.4 ± 1.2	0.64	.**008**
Total score (Qs 1-7)	41.4 ± 10.1	49.2 ± 7.4	0.64	.**008**
Q1 (facial appearance)	5.9 ± 1.5	7.3 ± 1.2	0.68	.**009**
Q2 (facial profile)	5.6 ± 2.0	7.0 ± 1.7	0.61	.**014**
Q3 (mouth's appearance)	5.8 ± 1.6	7.1 ± 1.4	0.66	.**007**
Q4 (rows of teeth)	5.9 ± 2.2	7.3 ± 1.4	0.53	.**026**
Q5 (shape/form of teeth)	6.0 ± 2.5	7.5 ± 1.7	0.50	.**029**
Q6 (color of teeth)	5.8 ± 2.6	5.5 ± 2.0	0.07	.662
Q7 (gum's appearance)	6.5 ± 2.0	7.5 ± 1.2	0.49	.**045**

*Note*: The values are reported as mean ± standard deviation. Statistically significant *P*-values are indicated in bold. A high score for DSCf suggests low dental self-consciousness (more favorable). Conversely, a high score for SC suggests a negative impact of the smile on social life, and a high score for DSCs suggests low dental self-confidence (less favorable).

### Personality Traits

The 2 study groups did not differ for any of the 5 personality traits assessed in the IPIP-NEO-60 survey.

### Correlation Analyses

Correlation coefficients between smile features, nasolabial AM scores, biomechanical properties of the cleft scar site, and self-perceived smile and facial esthetics as represented by the SERQoL and OES questionnaires are presented in Table 3.

**Table 3.** Correlation Matrix of Smile Features, Biomechanical Properties of the Cleft Scar, Perceived Facial Appearance and Smile QoL, and Nasolabial Scores.

	Smile features			Biomechanical properties of cleft scar site			Perceived facial appearance and smile QoL		
Cleft group	Frequency	Mean Duration	Rel. smile time %	Tone	Stiffness	Decrement	OES	DSCf	DSCs
Mean duration	0.42	−							
Rel. smile time %	0.58**	0.94**	−						
Tone	0.10	−0.08	0.01	−					
Stiffness	0.02	0.00	0.04	*0*.85**	−				
Decrement	−0.41	−0.44*	−0.49*	*−0*.*26*	*−0*.*05*	−			
OES	0.13	−0.04	0.02	−0.15	−0.41	−0.50*	−		
DSCf	0.32	0.14	0.20	−0.20	−0.46*	−0.53*	0.81**	−	
DSCs	−0.11	−0.18	−0.21	0.06	0.23	0.46*	−0.70**	−0.78**	−
Nasolabial AM scores	−0.38	−0.52*	−0.50*	*0*.*10*	*0*.*13*	*0*.*18*	−0.06	−0.19	−0.08

	Smile features			Biomechanical properties at “Cleft Scar”site			Perceived facial appearance and smile QoL	
Non-cleft group	Frequency	Mean duration	Rel. smile time %	Tone	Stiffness	Decrement	OES	DSCf
Mean duration	0.11	−						
Rel. smile time %	0.30	0.92**	−					
Tone	0.30	0.33	0.38	−				
Stiffness	0.31	0.05	0.08	*0*.91**	−			
Decrement	−0.33	−0.24	−0.18	*0*.*06*	*0*.*25*	−		
OES	0.02	0.32	0.35	0.12	−0.11	−0.38	−	
DSCf	0.01	−0.09	0.43*	0.10	0.11	0.15	0.43*	−
DSCs	−0.27	−0.58**	−0.64**	−0.51*	−0.30	0.13	−0.48*	−0.17

*Note*: Correlation coefficients between smile features and personality traits for the 2 study groups. Values represent Spearman correlation coefficients. *P*-values of statistical significance are indicated by asterisks (*p < .05; **p < .01). OES = Orofacial esthetics scale total score; DSCf = Dental self-consciousness; DSCs = Dental self-confidence.

In the cleft group, AM scores were negatively correlated with mean duration of smiles and relative smile time percentage, indicating that those with inferior nasolabial esthetic outcomes had a decreased tendency to smile. Decrement of the cleft scar was negatively correlated with mean duration of smiles and relative smile time percentage, indicating that non-elastic scars are associated with a reduced propensity to smile. Decrement was also negatively correlated with OES scores and DSCf scores, as well as positively correlated with DSCs scores, indicating that those with non-elastic cleft scars perceived their facial esthetics to be poor, are self-conscious of their smiles, and have low smile-related self-confidence. Stiffness of cleft scars was also negatively correlated with DSCf scores, indicating that those with stiffer scars felt more self-conscious of their smiles (Table 3).

In the non-cleft group, DSCs scores were negatively correlated with mean duration of smiles, relative smile time percentage, and lip tone at the equivalent “cleft scar” site; indicating that those who had low dental self-confidence had a reduced propensity to smile and had higher lip tonicity. DSCf scores were positively correlated with relative smile time percentage, indicating that those who were not self-conscious of their smiles had an increased propensity to smile. Social contact scores were not included in Table 3 as they were found to have no correlations with smile features, nasolabial AM scores, or biomechanical properties of the cleft scar site.

Correlations coefficients indicated in Table 4 show that within the cleft group, participants who were more extraverted felt more positive about their facial appearance (OES) and how their smile impacts their social life (SC). Participants in the cleft group who rated themselves poorly for facial appearance (OES) also rated themselves as having low dental self-confidence (DSCs) and felt that their smile negatively impacts their social life (SC)—the same correlation was observed in the non-cleft group for DSCs only.

**Table 4.** Correlation Matrix of Personality Traits and Perceived Facial Appearance and Smile QoL.

Cleft group	Personality traits					Perceived facial appearance and smile QoL	
	Neuroticism	Extraversion	Openness	Agreeableness	Conscientiousness	OES	DSCs
Extraversion	−0.54*	−					
Openness	−0.27	0.26	−				
Agreeableness	−0.26	0.05	−0.08	−			
Conscientiousness	−0.46*	0.38	−0.02	0.44*	−		
OES	−0.15	0.45*	0.27	0.06	−0.24	−	
DSCs	−0.02	−0.18	−0.13	−0.43	0.10	−0.70**	−
SC	0.24	−0.66**	−0.18	−0.24	−0.32	−0.54*	0.60**

Non-cleft group	Personality traits					Perceived facial appearance & smile QoL	
	Neuroticism	Extraversion	Openness	Agreeableness	Conscientiousness	OES	DSCs
Extraversion	−0.30	**–**					
Openness	0.17	0.07	**–**				
Agreeableness	−0.06	0.08	0.07	**–**			
Conscientiousness	−0.34	0.20	0.00	0.44*	**–**		
OES	−0.46*	0.26	0.01	0.08	0.44*	**–**	
DSCs	0.55*	−0.21	−0.22	−0.43	−0.49*	−0.48*	**–**
SC	0.36	−0.25	0.00	−0.01	−0.13	−0.30	0.69**

Abbreviations: OES, Orofacial esthetics scale total scores; DSCs, Dental self-confidence; SC, Social contacts.

*Note*:Correlation coefficients showing the personality traits and perceived smile and facial appearance for the 2 study groups. Values represent Spearman correlation coefficients. *P*-values of statistical significance are indicated by asterisks (*p < .05; **p < .01).

In the non-cleft group, the personality trait of neuroticism was negatively correlated with OES and positively correlated with DSCs, indicating that those with neurotic traits perceived their facial esthetics to be poor and had low dental self-confidence. Conversely, conscientiousness was positively correlated with OES and negatively correlated with DSCs, indicating that conscientious people perceive their facial esthetics favorably and have higher dental self-confidence. Dental self-consciousness (DSCf) scores were not included in Table 4 as no correlations were found with the personality traits.

Correlation coefficients for smile features and personality traits are presented in Table 5. In the cleft group, correlation coefficients showed that agreeableness was positively correlated with the mean duration of smiles (0.44; *p* < .05). In the non-cleft group, correlation coefficients showed that conscientiousness was positively correlated with mean duration of smiles (0.61; *p* < .01) and relative smile time percentage (0.57; *p* < .01), agreeableness was positively correlated with relative smile time percentage (0.46; *p* < .05), and openness was positively correlated with frequency of smiles (0.44; *p* < .05).

**Table 5.** Correlation Matrix of Smile Features and Personality Traits.

Cleft group	Smile features			Personality traits			
	Frequency	Mean duration	Rel. smile time %	Neuroticism	Extraversion	Openness	Agreeableness
Frequency	–						
Mean duration	0.42	–					
Rel. Smile Time %	0.58**	0.94**	–				
Neuroticism	0.11	0.06	−0.02	–			
Extraversion	0.10	0.16	0.26	−0.54*	–		
Openness	0.04	0.01	0.08	−0.27	0.26	–	
Agreeableness	0.03	0.44*	0.38	−0.26	0.05	−0.08	–
Conscientiousness	0.06	0.14	0.15	−0.46*	0.38	−0.02	0.44*

Non-cleft group	Smile features			Personality traits			
	Frequency	Mean duration	Rel. smile time %	Neuroticism	Extraversion	openness	Agreeableness
Frequency	–						
Mean duration	0.11	–					
Rel. smile Time %	0.30	0.92**	–				
Neuroticism	−0.12	−0.34	−0.25	–			
Extraversion	0.28	−0.02	0.06	−0.30	–		
Openness	0.44*	0.14	0.41	0.17	0.07	–	
Agreeableness	0.41	0.35	0.46*	−0.06	0.08	0.07	–
Conscientiousness	0.10	0.61**	0.57**	−0.34	0.20	0.00	0.44*

### Video Ratings

Participants in the non-cleft group found the video more amusing, (mean rating 7.2. ± 1.6 out of maximum of 10) compared to the cleft group (mean rating = 6.2 ± 1.4), the difference being statistically significant (*p* < .05).

## Discussion

### Smile Features

The primary aim of this study was to investigate the impact of unilateral cleft lip with or without cleft palate on smile behavior using 2 distinct groups—1 group consisting of participants who had undergone treatment for a UCL ± P, and the other a non-cleft control group. This involved investigating an individual's inclination to smile, measured by variables such as smile frequency, duration, and percentage of time spent smiling; as well as the characteristics of smiles, including smile intensity, the amount of tooth show indirectly measured by the distance between the lips, and the genuineness of smiles.

No significant differences for any of the smile outcomes assessed were found between the 2 study groups. This unexpected result contradicts our study hypothesis, which anticipated reduced smile frequency, duration, intensity and tooth display in the cleft group. Furthermore, as the cleft group participants rated our video as being less amusing compared to the control group, we would have expected them to smile even less. Therefore, the variation in video ratings does not explain the lack of difference in smile features.

Our research findings contrast with previous subjective accounts of reduced smiling in individuals with CL/P, suggesting that their objectively measured smile behavior is comparable to that of the general population. This somewhat affirms recent systematic reviews that have indicated a generally low overall impact of CL/P on psychological outcomes and adjustment.^4,33^

Although these positive findings may appear encouraging, it is crucial to acknowledge that our study was carried out in laboratory settings, which do not replicate real-life social situations and therefore have limited external validity. Participants viewed the videos alone, potentially lacking the same sense of self-consciousness about smiling that can arise in social contexts. Additionally, the sample size was limited to detecting a meaningful difference of 50% in the frequency of smiling between the groups, potentially hampering the detection of smaller variations.

It is also important to acknowledge that our results are more applicable to individuals with unilateral clefts involving both the lip and the palate, as most participants in this study were diagnosed with UCLP, and only 4 out of the 21 participants were diagnosed with UCL. A limitation of our study is the lack of separate analysis for these 2 diagnoses, therefore preventing us from determining the impact of UCL compared to UCLP on smiling behavior. This distinction is important as existing literature suggests there is variation in certain psychological outcomes based on cleft type and treatment variables.^4,6^ We would expect the impact of each diagnoses to differ, given that surgical procedures and resulting scar tissue vary between the 2, with UCLP typically involving a more extensive surgical course.

Interestingly, our findings contradict with a recent study that was conducted using the same smile analysis method.^24^ In that study, participants with severe malocclusions and participants who had completed orthodontic treatment 1 year prior for severe malocclusions, smiled significantly less compared to a control group. Given that the severe malocclusion group still had reduced tendency to smile post-treatment, we would have expected to also find this amongst the cleft group participants in our study. A possible explanation could be that the majority of cleft group participants in our study had well surpassed one year following their treatment. This extended timeframe might have allowed for their smile behavior to adapt and normalise.

With the above limitations in mind, our unexpectedly positive smile results offer a nuanced perspective that enriches the existing literature. Future studies investigating cleft smiles may benefit from analysing additional variables such as cleft type, treatment, age, sex, and socioeconomic status, as these factors show variability in psychological outcomes.^6,8,33,34^

### Clinical & Biomechanical Outcomes and Correlations

The secondary objective of our study was to explore the potential influence of a clinical outcome, namely nasolabial AM scores; biomechanical properties of the lips; and psychosocial factors, including self-perceived Smile Esthetics-related Quality of Life, Orofacial Esthetics, and personality, on an individual's smiling features.

### Nasolabial AM Scores

Nasolabial AM scores, were exclusively assessed within the cleft group, providing an assessment of the esthetic outcomes of cleft treatment. The main objective for collecting this data was to investigate its influence on an individual's propensity to smile, specifically within the cleft group. Consistent with our hypothesis, it was found that poor nasolabial esthetics was associated with a decreased propensity to smile, as indicated by statistically significant correlations with 2 smile variables—mean duration of smiles and relative smile time percentage. The nasolabial data demonstrated excellent and good reliability within and between raters, establishing its reliability as a measurement in this study.

There were no identified correlations between nasolabial esthetics scores and participants’ self-perception of their facial esthetics. This finding is consistent with other studies that have also reported a lack of significant correlation between professionally examined nasolabial appearance and patient self-assessment.^35,36^ This reaffirms the importance of integrating patient-reported outcomes with clinically assessed measures, both in the clinical setting and in future research.

While we discuss comparisons for the AM scores, we urge caution when interpreting these results. The values obtained in our study are not representative of the current esthetic outcomes for individuals with UCLP in New Zealand, and more accurate reports can be found in other studies.^37^ This limitation arises from several factors: firstly, our study included participants of a wide age range, secondly, our sample included participants with diagnoses of both UCLP and UCL, and thirdly, 2 of these participants had their cleft treatment partially or entirely completed overseas. Additionally, it is worth noting that our panel of nasolabial examiners consisted solely of female dental specialists. It has been shown that individuals of different genders and levels of expertise reach different nasolabial scores.^38^ Consequently, direct comparisons to other studies that have employed larger panels with a wider range of examiner expertise and genders may not be accurate.

Nevertheless, the mean nasolabial score obtained from our study was 2.5 ± 0.6, indicating overall fair to very good esthetic outcomes. These results are similar to a nationwide study in New Zealand from 2018, and are slightly better than the results from the Americleft and Eurocleft studies.^37,39,40^ However, the participants in our study were significantly older than the studies above, and there is considerable time has passed since the Americleft and Eurocleft studies. A more relevant comparison may be made with a study conducted in Austria that assessed nasolabial esthetics from photos of individuals aged 15 to 30 years old who had completed treatment for a UCLP.^38^ The majority of participants from our study were born in the 1990s, and when compared to the Austrian study participants who were also born in the 1990s, our nasolabial results appear marginally worse.

### Biomechanical Properties of the Lips and Cleft Scar

To the best of our knowledge, our study is the first to use myotonometry for evaluating the biomechanical properties of perioral muscles in a sample of individuals with cleft. Notable differences were found in the muscle characteristics of the cleft group participants when compared to the control group.

At 2 adjacent locations, the cleft scar and the central upper lip, elevated levels of muscle tone and stiffness were observed. These sites were in close proximity to each other, and in certain participants, the cleft scar extended into the boundaries of the central upper lip. Consequently, there is a strong likelihood that both these locations are indicative of the pathological characteristics of the cleft scar. Scar tissue displays an abundance of irregularly arranged collagen fibres, which could potentially account for the increased stiffness and tone observed.^41^ These findings also support previous research, which has found greater stiffness of the overall perioral musculature in patients with a repaired cleft who have not had revision surgeries, as well as increased stiffness on the philtrum ridge of the cleft side in patients with repaired UCLP.^17,42,43^

Additionally, higher decrement, indicating reduced elasticity, was observed at the left upper lip, with a tendency for the same at the right upper lip. While this difference was only significant on the left side, it is reasonable to hypothesise that this was attributed to majority of participants in our study having left-side clefts. It is possible that those with right-side clefts exhibited lower elasticity at the upper right lip, however, right and left-side clefts were not analysed separately during our investigation.

It is interesting that we found a pattern of increased tone and stiffness at the cleft scar, while decreased elasticity was only observed adjacent to the scar. This decline in elasticity of the adjacent tissue may be attributed to a reduction in the total thickness of the upper lip, which tends to be taut and flattened in individuals with repaired cleft lips due to a decrease in overall lip volume.^43,44^ This finding partially aligns with previous research, which identified decreased elasticity, along with increased tone and stiffness, in thin lips.^27^ Although there was also a tendency for increased tone at the left upper lip, it did not reach statistical significance. It is possible, however, that our sample size was insufficient to detect additional statistically significant differences in the outcomes with sufficient power.

Furthermore, insightful correlations between the functional characteristics of cleft scars and their impact on smiling outcomes have been demonstrated. Correlation coefficients have revealed that the elasticity of the cleft scar within the cleft group is a significant factor affecting smile features, as well as the perceived facial appearance and smile esthetics-related quality of life. Within the cleft group, elasticity of the cleft scar was positively correlated with duration of smiles and relative smile time, and associated with poorer (higher) scores for self-perceived orofacial esthetics, dental self-consciousness, and dental self-confidence. Additionally, stiffer cleft scars were associated with poorer dental self-consciousness.

None of these correlations were found within the control group. The only biomechanical correlation found within the control group was that of higher tone at the equivalent “cleft scar” site, being associated with increased dental self-confidence. It is unlikely that this stand-alone correlation is of any clinical significance, and it is possible that it may have occurred by chance as a false positive.

These observed correlations align with our initial hypothesis and suggest that functional attributes of cleft scars, particularly reduced elasticity, can have a detrimental effect on an individual's inclination to smile. These findings emphasise the significance of clinically evaluating scar elasticity and highlight the need for interventions aimed at improving functional scar outcomes for individuals with UCL ± P.

### Psychosocial co-Variates and Correlations

Participants in the cleft group displayed significantly lower scores compared to the non-cleft group for Smile Esthetics-related Quality of Life. Specifically, they reported lower scores in the areas of social contacts, indicating that they feel their smile negatively impacts their social life, as well as in dental self-confidence.

To our knowledge, no other studies have utilised the SERQoL survey in a cohort of participants with cleft conditions. Nevertheless, our findings align with existing research, which has demonstrated that adults affected by CL/P conditions experience diminished health-related quality of life, particularly in the domains of social functioning and emotional role, as well as reduced oral health-related quality of life.^2,3,10^

Furthermore, the cleft group participants rated their orofacial appearance significantly lower than the non-cleft group across 6 out of 7 assessed features, with lower total and overall scores on the Orofacial Esthetic Scale. These results highlight the challenges faced by individuals in the cleft group regarding their self-perception of orofacial appearance and underscore the impact of cleft lip conditions on overall esthetic satisfaction.

Contrary to our hypothesis, there was no apparent link between the way participants in the cleft group perceived their facial appearance and smile esthetics-related quality of life, with their inclination to smile. No correlations were found in the cleft group between OES and SERQoL scores, with smile outcomes. However, in the non-cleft group, elevated levels of dental self-confidence were associated with a higher percentage of time spent smiling.

Interestingly, among individuals with a cleft, the personality trait of extraversion was linked to a more positive evaluation of their orofacial appearance and a greater sense of satisfaction regarding how their smile influences their social interactions. Additionally, agreeableness was correlated with extended duration of smiles.

In the non-cleft group, neuroticism emerged as an unfavorable personality trait, as it was associated with poorer self-perceived orofacial esthetics and low dental self-confidence. In contrast, several positive (favorable) traits were observed: conscientiousness exhibited positive correlations with both the duration of smiles and the percentage of time spent smiling, agreeableness displayed a positive correlation with the percentage of time spent smiling, and openness showed a positive correlation with the frequency of smiles.

While personality traits were not the focus of this study, they offer another perspective to consider and emphasise the significance of tailoring treatment goals to individual patients. Our research also reinforces the recommendation for continuous psychosocial support, even after completing cleft care, based on the low scores observed in the cleft group regarding self-perceived smile esthetics-related quality of life and orofacial esthetics.

As a final note, it must be acknowledged that our results are not fully representative of the New Zealand population as, despite efforts being made to encourage Māori participation, unfortunately no individuals identifying as Māori applied. However, our study was successful in achieving participation from people throughout the country and of a wide age range. Furthermore, our study findings may be somewhat generalisable and useful for people affected by other types of CL/P conditions and craniofacial anomalies.

## Conclusion

Automated analysis of smile episodes showed no differences in smile features amongst adolescents and adults who had undergone treatment for a UCL ± P, in comparison to a control group. This indicates that people with UCL ± P exhibit similar smile behavior as the general population—at least in non-social settings. However, unsatisfactory nasolabial AM scores and low elasticity of the cleft scar were found to be linked to a diminished tendency to smile. As a result of this research, we have demonstrated that myotonometry is a valid clinical tool to objectively assess the physical properties of cleft scars and the perioral musculature. Interestingly, while psychosocial factors, including self-perceived smile esthetics-related quality of life and orofacial esthetics, were notably negatively impacted within the UCL ± P group, they did not demonstrate any association with smile outcomes. In light of these lower self-reported factors, we advocate for the provision of continuous psychosocial support for adults affected by UCL ± P.

## Figures and Tables

**Appendix 1.**
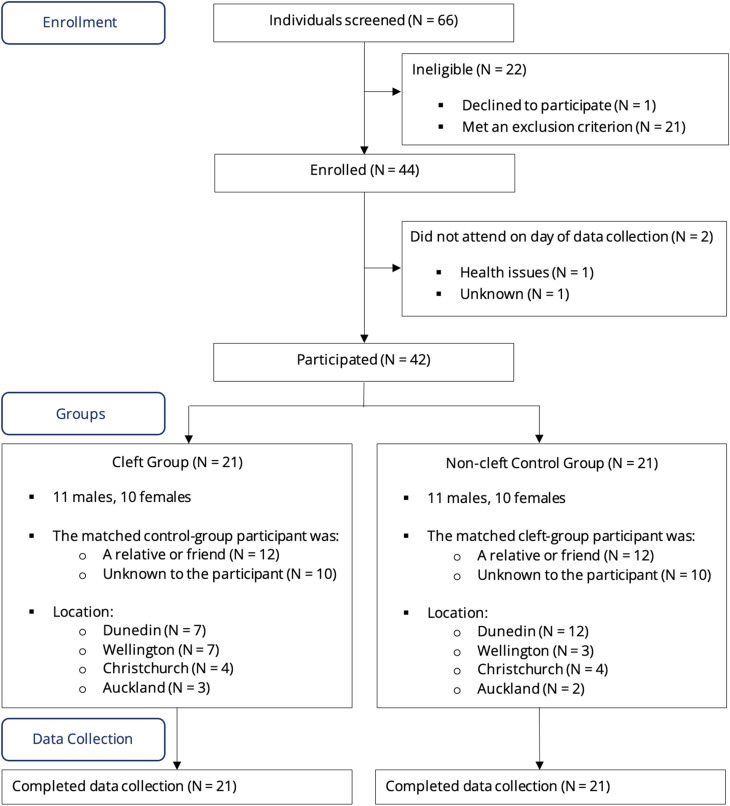
A flowchart showing the recruitment process.

**Appendix 2.**
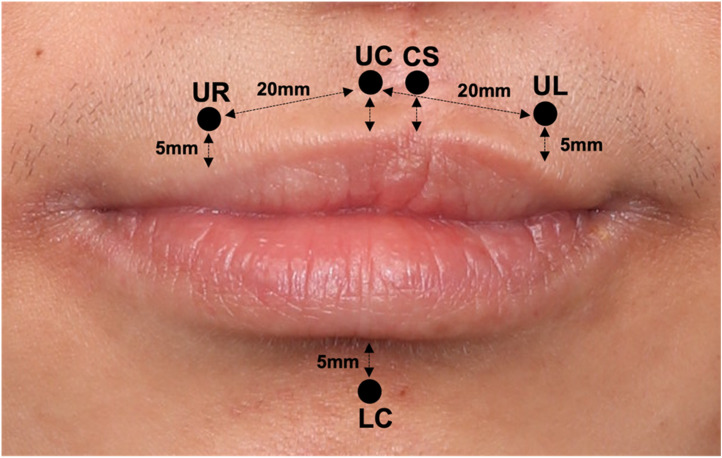
Perioral landmarks of interest in a participant with left-side cleft lip. The cleft scar (CS) point was located on the midpoint of the cleft scar and 5 mm superior to the vermillion border of the upper lip. For the non-cleft group participants, CS was located on the ipsilateral equivalent spot as the matched cleft group participant, on the philtrum ridge, and 5 mm superior to the vermillion border. The upper central (UC) point was located 5 mm superior to the midline of the vermillion border. The upper right and left (UR and UL) points were located 5 mm superior to the vermillion border and 20 mm to the right and left of the midline, respectively. The lower central (LC) point was located 5 mm inferior to the midline of the vermillion border of the lower lip.

**Appendix 3.**
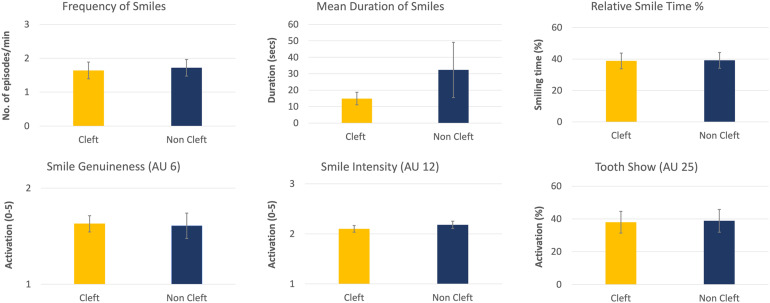
Bar graphs showing the smile features of the 2 study groups investigated. The first row contains the frequency of smiles, mean duration of smiles, and the relative smile time smile (*N* = 42). The second row contains the activation of the 3 action units (AU6, AU12, AU25); representing smile genuineness, smile intensity, and tooth show (*n* = 38). Bars represent estimated marginal means, whereas error bars represent the standard errors of mean.

**Appendix 4.**
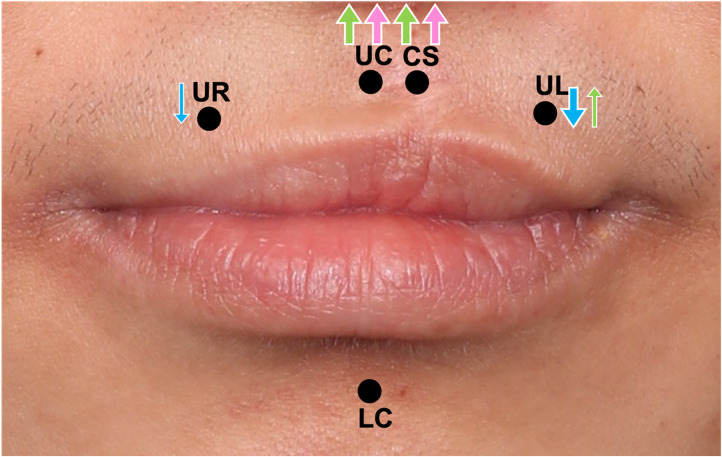
Biomechanical properties of the lips in the cleft group compared to the non-cleft group are represented by arrows at each site. Higher tone (green) and stiffness (pink) were found at the upper central and cleft scar sites. Lower elasticity (blue) was found at the upper left and right sites, as well as higher tone at the upper left site. Thick arrows represent differences of strong statistical significance (*p* < .001), whereas thin arrows represent differences of marginal statistical significance (*p* > .05).
